# Scopolamine Reduces Electrophysiological Indices of Distractor Suppression: Evidence from a Contingent Capture Task

**DOI:** 10.3389/fncir.2017.00099

**Published:** 2017-12-06

**Authors:** Inga Laube, Natasha Matthews, Angela J. Dean, Redmond G. O’Connell, Jason B. Mattingley, Mark A. Bellgrove

**Affiliations:** ^1^Queensland Brain Institute and School of Psychology, The University of Queensland Brisbane, QLD, Australia; ^2^ImpAct Team, Lyon Neuroscience Research Center, INSERM U1028, CRNS-UMR5292 Lyon, France; ^3^School of Psychological Sciences and Monash Institute of Cognitive and Clinical Neurosciences, Monash University Melbourne, VIC, Australia; ^4^Trinity College Dublin, Trinity College Institute of Neuroscience Dublin, Ireland

**Keywords:** attention, acetylcholine, muscarinic, ERP, feature, N2pc, Pd

## Abstract

Limited resources for the in-depth processing of external stimuli make it necessary to select only relevant information from our surroundings and to ignore irrelevant stimuli. Attentional mechanisms facilitate this selection via top-down modulation of stimulus representations in the brain. Previous research has indicated that acetylcholine (ACh) modulates this influence of attention on stimulus processing. However, the role of muscarinic receptors as well as the specific mechanism of cholinergic modulation remains unclear. Here we investigated the influence of ACh on feature-based, top-down control of stimulus processing via muscarinic receptors by using a contingent capture paradigm which specifically tests attentional shifts toward uninformative cue stimuli which display one of the target defining features In a double-blind, placebo controlled study we measured the impact of the muscarinic receptor antagonist *scopolamine* on behavioral and electrophysiological measures of contingent attentional capture. The results demonstrated all the signs of functional contingent capture, i.e., attentional shifts toward cued locations reflected in increased amplitudes of N1 and N2Pc components, under placebo conditions. However, scopolamine did not affect behavioral or electrophysiological measures of contingent capture. Instead, scopolamine reduced the amplitude of the distractor-evoked Pd component which has recently been associated with active suppression of irrelevant distractor information. The findings suggest a general cholinergic modulation of top-down control during distractor processing.

## Introduction

Selective attention allows adaptive processing of sensory input via top-down control signals, which modulate stimulus-related activity in sensory cortex in favor of behaviorally relevant stimuli (Serences et al., 2005; Liu et al., 2007; Jehee et al., 2011; Lustig and Beck, 2012). As a result, the cortical representation of behaviorally relevant stimuli is enhanced relative to irrelevant stimuli facilitating their selection for further processing (Fecteau and Munoz, 2006; Serences and Yantis, 2007). Attentional control of stimulus processing is thought to be influenced by several neuromodulators (Noudoost and Moore, 2011). Prominent among these is acetylcholine (ACh), which has been shown to optimize stimulus processing in attention demanding contexts (Sarter et al., 2005). It remains unknown, however, if this role is limited to spatial attention or if it also extends to feature-based aspects of attention.

ACh exerts its modulatory role over attentional processes via two different receptor types, namely muscarinic and nicotinic ACh receptors. These receptor types have different distributions in the brain but also appear to influence distinct aspects of attentional control (Zilles et al., 2002). Whereas nicotinic ACh receptors are thought to be more involved in novelty and cue detection, muscarinic receptors recruit circuits required for top-down control of attention (Hasselmo and Sarter, 2011). Evidence for the latter stems from spatial cueing studies in monkeys (Davidson et al., 1999; Davidson and Marrocco, 2000) and humans (Dunne and Hartley, 1986), which suggest that the muscarinic antagonist *scopolamine* might reduce the optimal utilization of attentional resources at a cued location. In line with this premise, an electrophysiological study in monkeys (Herrero et al., 2008) demonstrated a modulatory effect of scopolamine on spatial attention in visual cortex. However, despite the strong indications of a predominantly muscarinic modulation of top-down attention, there has been only limited research in human participants addressing this issue. Although some studies have confirmed muscarinic effects in visual cortex using neuroimaging methods (Furey et al., 2000; Mentis et al., 2001), the poor temporal resolution of neuroimaging means that these studies are uninformative with respect to the temporal cascade of information processing that contributes to attentional modulation. To our knowledge, no studies have utilized the excellent temporal resolution of event related potential (ERP) recordings to investigate cholinergic effects during different stages of visual processing in the human brain.

In this study we asked whether previous findings of cholinergic modulation of top-down attentional control, which focused on spatial attention, can also be generalized to the feature-based aspect of top-down control. Feature-based attention enhances the representation of stimuli with attended features and thus optimizes the search for a target based on target-defining features in the context of spatial uncertainty. This effect can be measured using contingent capture paradigms, in which attention involuntarily shifts toward a spatial cue when the cue contains a feature that matches the current task set (e.g., the color *red*; Folk et al., 1992). As a result, cues matching the task set induce a validity effect, which is characterized by faster detection of targets appearing at cues locations compared to targets in other locations. Together with the electrophysiological characterization of the contingent capture effect this paradigm represents an excellent tool for the measurement of feature-based attention (Folk et al., 1994; Ansorge and Heumann, 2003; Ansorge et al., 2005; Chen and Mordkoff, 2007; Leblanc et al., 2008; Lien et al., 2008; Brisson et al., 2009; Sawaki and Luck, 2013). We therefore investigated whether the muscarinic antagonist *scopolamine* could affect behavioral and electrophysiological markers of contingent capture.

To assess the influence of scopolamine on contingent capture we focused analysis on several distinct electrophysiological markers. First, to investigate early processing of visual stimuli, we focused on the visual P1 and N1 components. Both of these components have been shown to be the earliest components modulated by top-down attention, including feature-based attention (Mangun and Hillyard, 1991; Lange et al., 1998; Arnott et al., 2001; Hopf et al., 2004; Zhang and Luck, 2009; Luck and Kappenman, 2012). Second, we were interested in components reflecting attentional orienting and distractor suppression, namely the N2pc and Pd. The N2pc is a reliable indicator of spatially selective processing of lateralized, task-relevant visual stimuli when these are presented with distractors (Luck and Hillyard, 1994a,b; Eimer, 1996; Woodman and Luck, 1999; Eimer and Kiss, 2008; Kiss et al., 2008; Luck, 2012). The N2pc has been used in several studies to demonstrate contingent capture (Eimer and Kiss, 2008, 2010; Leblanc et al., 2008; Eimer et al., 2009). Third, we investigated the Pd (posterior positivity) component, which is closely related to the N2pc component, often follows the N2pc and is involved in distractor suppression (Sawaki and Luck, 2011, 2013; Hilimire et al., 2012; Kiss et al., 2012; Sawaki et al., 2012; McDonald et al., 2013).

We hypothesized that modulation of top-down control of attention should be reflected in the modulation of one or several of these components and the nature of the modulation should thus speak to the role of muscarinic modulation of top-down control.

In light of previous studies of the muscarinic modulation of attention, we predicted that scopolamine would impair the processing advantage for task-relevant features and would therefore reduce the capturing effect of target-colored cues. This effect should be evidenced by a reduction of the validity effect as well as reduced amplitude, or latency shift, in the cue-related N2pc and/or Pd component. We also expected to observe a change in the amplitude of the P1 and N1 related to target-colored cues. Under placebo conditions, the P1 and N1 amplitude for these cues should be increased compared with neutral cues, due to top-down feature-based modulation. Thus, an inhibitory effect of scopolamine on top-down feature-based attention should reduce the amplitude of target-colored cues. To our knowledge, this study represents the first attempt to dissect specific effects of scopolamine on feature-based top-down attention using electrophysiological recordings.

## Materials and Methods

### Participants

Thirty male Caucasian participants with normal or corrected-to-normal vision and no history of neurological or psychiatric illness were tested. Data from two participants was excluded from the analysis due to poor performance, and a further one dataset due to equipment failure during EEG recording. Of the remaining 27 participants (mean age 27.9 ± 6.1 years), 25 were right-handed and two were left-handed according to results from the Edinburgh Handedness Inventory (Oldfield, 1971). Participants gave written informed consent in accordance with the Declaration of Helsinki. The study was approved by the University of Queensland’s ethics committee.

All participants underwent a rigorous clinical interview to exclude confounding conditions such as major psychiatric illness, neurologic illnesses or drug dependency.

### Study Design

The effects of scopolamine on attention were tested using a double-blind, placebo-controlled, cross-over design with two acute treatment sessions. Sessions were separated by a 7-day washout period and testing took place at the same time of day in both sessions. At the start of each session two gelatine capsules containing 0.8 mg scopolamine or dextrose (Placebo) were ingested with water. The dose for Scopolamine was chosen based on previous publications, which together suggest reliable cognitive effects but minimal sedative effects on performance at this dose of the drug (Ostfeld et al., 1959; Safer and Allen, 1971; Wood et al., 1985). Participants performed the contingent capture task from 100 (±15) min to 140 (±15) min after drug administration, coinciding with peak plasma levels of the drug (Muir and Metcalfe, 1983; Wood et al., 1985). Side effects were assessed before drug administration, at drug peak and after the contingent capture task had been performed, by measuring blood pressure and heart rate as well as using a visual analog scale (VAS) for subjective side effect ratings (alertness, contentedness and calmness; Bond et al., 1974).

General effects of scopolamine on arousal were tested in a two-alternative forced choice reaction time task, in which participants gave speeded responses to the direction (left, right) of a central arrow using the left and right index finger. This task was administered before drug administration and just before testing with the contingent capture task.

### Setup and Stimuli

**Figure 1:**
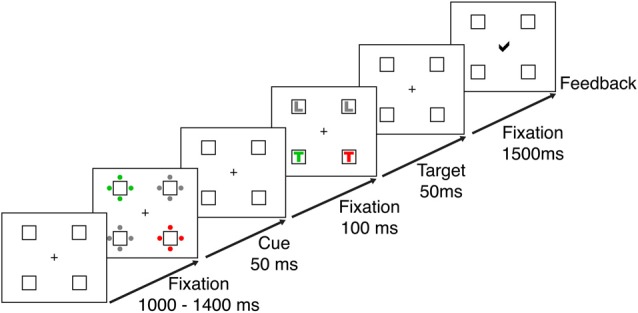
Trial sequence for the contingent capture task. Each trial started with a fixation display, which was presented for a randomized interval of 1000–1400 ms. Cue and target displays appeared for 50 ms each and were separated by a 100 ms interval, during which the fixation display was presented. After the targets disappeared, participants had 1500 ms to respond to the target identity. At the end of the trial participants received visual feedback about the accuracy of their responses.

Stimulus presentation and recording were implemented using Presentation^®^ software (Version 14.1)^1^ and a 21″ CRT monitor with a screen resolution of 1024 × 768 pixels at a viewing distance of 70 cm. Responses were recorded using a standard keyboard. Participants were tested in a contingent capture reaction time task adapted from Lien et al. (2008). The task required speeded responses to the identity of a letter (L or T) shown in a predefined target color (red or green). Responses were made using the index fingers of the left and right hands. The letter corresponding to each response button was counterbalanced between participants. The target-colored letter appeared with equal probability in one of four placeholder boxes located at the corners of an imaginary square surrounding a central fixation cross on a black background. The boxes subtended 1.8 degrees visual angle and were displayed at a distance of 2.8 degrees from central fixation. The remaining boxes of the target display contained distractor letters, one in a non-target color and two in gray (see Figure 1). The target display was preceded by a cue display, which contained four sets of four dots arranged around the placeholder boxes. Depending on the cue condition the sets of dots were presented in different colors (see Figure 1 and below). The fixation display consisted of the central fixation cross and the four placeholder boxes. RGB values of all colors were measured using a chromameter (Konica Minolta CL-100), and were adjusted to ensure equiluminance between all stimuli.

### Task Design

The display sequence for a single trial is shown in Figure 1. At the beginning of each trial the central fixation cross blinked to ensure central fixation at trial onset. After a randomized delay of 1000–1400 ms participants were presented with the cue display, which was followed by the fixation display again and then the target display. Cue and target displays were on the screen for 50 ms each, and were separated by an inter-stimulus interval of 100 ms, during which the fixation display was presented.

The target display was followed by another fixation display, which lasted until a response was given but no longer than 1500 ms. Responses faster than 200 ms or slower than 1500 ms after target onset were not registered. At the end of each trial participants received visual feedback on the accuracy of their responses (correct, incorrect), displayed at the center of the screen. For incorrect responses the visual feedback was accompanied by a 1000 Hz tone.

**Figure 2:**
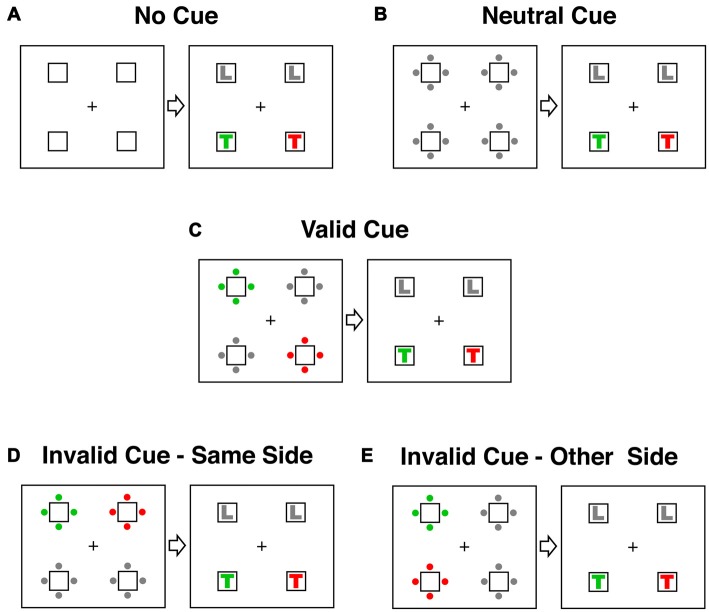
Cue and target conditions in the contingent capture experiment. Responses were classified according to the spatial relationship between cue and target events. In the examples displayed here the target color is RED and the distractor color is GREEN. **(A)** The no-cue condition did not have a cue display. Instead the target appeared 1150–1550 after trial onset. **(B)** In the neutral condition all dots in the cue display were gray. **(C)** In the valid condition the target-colored (red) dots in the cue display appeared in the same location as the following target letter (red T). **(D)** In the “invalid-same-side” condition the target-colored cue appeared on the same side (left or right) as the target letter but at a different location (above or below). **(E)** In the “invalid-other-side” condition the target-colored cue appeared on the opposite side of the display to the target letter.

Five different stimulus categories representing all possible spatial relationships between cue and target were tested (see Figure 2). In the three main conditions (valid, invalid-same-side and invalid-other-side) one set of cue-dots had the target color, one set the distractor color and two sets were gray. In the *valid cue condition* (Figure 2C) the target-colored dots in the cue display appeared in the same location as the target-colored letter in the target display. In both of the invalid conditions the target-colored letter appeared at a different location to the target-colored cue. To account for attention shifts within and between the two sides of the stimulus display, we distinguished between an invalid-same-side condition (Figure 2D) and an invalid-other-side condition (Figure 2E). In the *invalid-same-side condition* the target-colored cue appeared on the same side (left or right) but at a different spatial location to the target. In the *invalid-other-side condition* the target-colored cue appeared on the side opposite to the target.

There were also two “baseline” conditions. A *no-cue condition* allowed assessment of general drug effects on arousal. In this condition (Figure 2A) the target was presented at the same latency as in the other four testing conditions (1150–1550 ms after trial onset) but was not preceded by a cue. In the *neutral cue condition* all dots in the cue display were gray (Figure 2B). This condition allowed a comparison of the general alerting effects of the cue with the specific spatial cueing effects of the valid and invalid trials. All conditions were equiprobable and the presentation of all conditions was fully randomized.

Reaction times (RTs) and accuracy were analyzed using Matlab (Version 7.13, The MathWorks, Inc.) and SPSS (Version 20, SPSS, Inc.). Trials with RTs above or below three standard deviations from the condition mean per participant were excluded from the analysis.

### Procedure

In each session participants completed one run with the target color as red and one run with the target color as green, with the order counterbalanced between participants. At the beginning of each testing session, and when switching to a different target color, participants completed a block of 20 practice trials. Every run consisted of six blocks of 48 trials, each separated by enforced rest breaks of 1 min. Each testing condition comprised 48 trials per run with fully randomized presentation across testing blocks, resulting in 96 trials for each condition per participant and drug condition. Cue and target position within each condition were counterbalanced within each run.

### EEG Recording and Analysis

Continuous EEG data were recorded using an ActiveTwo Biosemi electrode system from 64 scalp electrodes, digitized at 1024 Hz. Vertical eye movements were recorded with two vertical electrooculogram (EOG) electrodes above and below the left eye, while horizontal eye movements were recorded from electrodes placed at the outer canthus of each eye. Data were analyzed using eeglab 9.0.4.4. (Delorme and Makeig, 2004) and FASTER v1.2b. (Nolan et al., 2010), implemented in Matlab.

The resulting EEG data were down-sampled to 512 Hz, re-referenced off-line to the average of all scalp electrodes and segmented into epochs of −1000 to +2000 ms surrounding cue onset. Epochs were baseline corrected relative to the pre-cue interval (−200 to 0 ms), high-pass filtered to 0.5 Hz and low-pass filtered up to 40 Hz. To eliminate blink and eye movement artifacts an independent components analysis was conducted using FASTER v1.2b. (Nolan et al., 2010) and the respective components eliminated from the signal of all channels. Finally, the signals in all epochs were checked manually and epochs with extreme transient noise were rejected.

In the subsequent analyses grand averages were generated for the calculation of the N1, P1, N2pc and Pd components. Peak amplitudes and mean amplitudes were analyzed using a region of interest (ROI) approach. Electrode sets for all analyses were selected based on the peak voltage of each component in a topographic map in the placebo condition and guided by previous reports of each component in the literature. The width of the window used to measure component amplitudes was based on the duration and spatial extent of each component in the grand average waveform. Peak amplitudes were extracted as the maximum voltage for positive components or the minimum voltage for negative components within the respective time window. The mean amplitude was calculated as the average voltage within the time window for each component. Peak latencies, i.e., the timepoint of the maximum amplitude, were measured in relation to the onset of the respective stimulus display (target or cue).

The following intervals were used to determine peak amplitude or mean amplitude measures: P1 80–120 ms, N1 150–200 ms, cue-N2pc 200–250 ms, Pd 275–325 ms and target-N2pc 350–450 ms after cue onset.

Signals were averaged over electrodes PO3, PO4 and POz for the analysis of drug effect on the cue-related P1 component. The choice of electrodes was based on inspection of scalp voltage topography under placebo conditions in the time window of +80 to 120 ms after cue onset (see Figures 4A,B).

**Figure 3:**
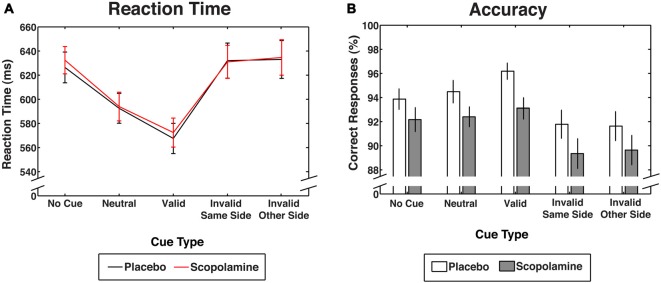
Performance measures for contingent capture under placebo and scopolamine conditions. **(A)** Reaction times (RTs) in milliseconds (ms) for each cue condition. **(B)** Accuracy as percentage (%) of correct responses for each cue condition. All measures are displayed as group means with ± 1 standard error; *n* = 27.

**Figure 4:**
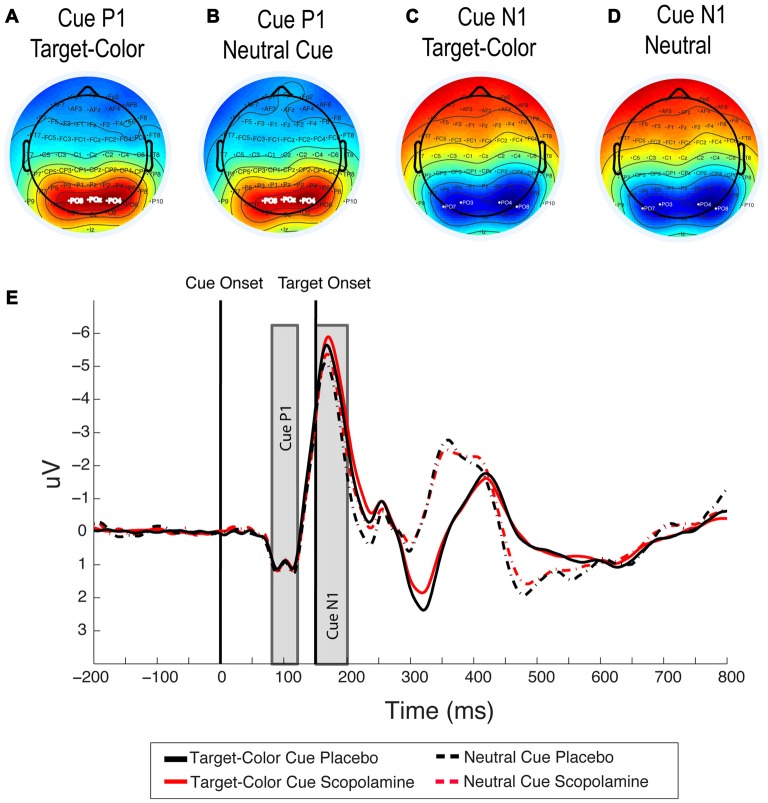
**(A,B)** Topography of the cue-related P1 component under placebo for target-colored cues and neutral cues averaged between 80 ms and 120 ms after cue onset. **(C,D)** Topography of the cue-related N1 component under placebo for target-colored cues and neutral cues, averaged between 150 ms and 200 ms after cue onset. **(E)** Grand-average waveforms illustrating cue related P1 and N1 components for neutral and target-colored cues, under placebo and scopolamine, averaged over PO3, PO4 and POz electrodes for the P1 component and over PO7, PO3, PO8 and PO4 electrodes for the N1 component; *n* = 27.

For the analysis of drug effect on the cue-related N1 component we averaged signals over electrodes PO7, PO3, PO8 and PO4. The choice of electrodes was based on inspection of scalp voltage topography under placebo conditions in the time window of +150 to 200 ms after cue onset (see Figures 4C,D). In this analysis all trials with color cues were pooled into one grand average and compared to the grand average of the neutral cue condition.

**Figure 5:**
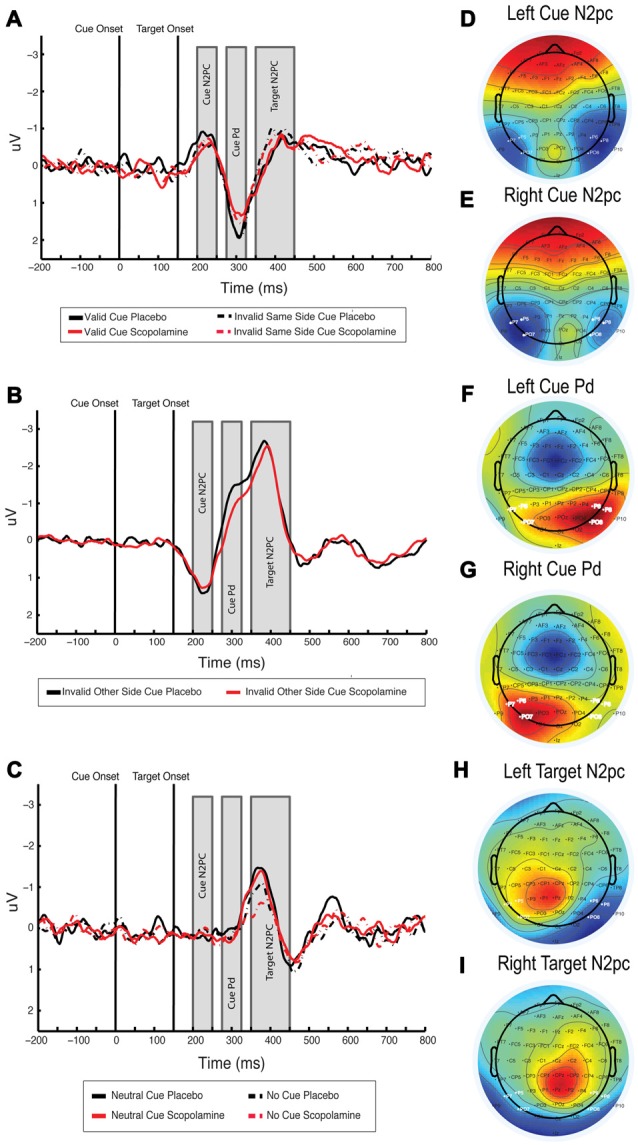
**(A)** Difference waveform illustrating cue N2pc, cue Pd and target N2pc for invalid-same-side and valid cues, waveforms averaged over PO7/P7/P5 and PO8/P8/P6, respectively, for left and right and then collapsed over side. **(B)** Difference waveform illustrating cue N2pc, cue Pd and target N2pc for invalid-other-side cues, waveforms averaged over PO7/P7/P5 and PO8/P8/P6, respectively for left and right and then collapsed over side. Note that waveforms were calculated with respect to the target location, resulting in a positive going cue N2pc. **(C)** Difference waveform showing cue N2pc, cue Pd and target N2pc for neutral and no-cue conditions. Waveforms are averaged over PO7/P7/P5 and PO8/P8/P6, respectively, for left and right and then collapsed over side. **(D,E)** Topography of the cue-related N2pc component under placebo for left and right target-colored cues, averaged between 200 ms and 250 ms after cue onset. **(F,G)** Topography of the cue-related Pd component under placebo for left and right target-colored cues, averaged between 275 ms and 325 ms after cue onset. **(H,I)** Topography of the target-related N2pc component under placebo for left and right targets, averaged between 200 ms and 300 ms after target onset; *n* = 27.

For the analysis of N2pc and Pd components, we selected electrodes based on the scalp voltage topography for target-colored cues under placebo conditions in the time window of +200 to 250 ms after cue onset (see Figure 5). We chose the electrodes PO7, P7, P5 in the left hemisphere and PO8, P8, P6 in the right hemisphere for the calculation of the cue N2pc. Mean amplitudes of N2pc and Pd were calculated from the difference waveforms derived from the subtraction of signal from contralateral and ipsilateral electrodes. The difference waveforms for targets were calculated with respect to the target location (e.g., left target = right electrodes minus left electrodes; right target = left electrodes minus right electrodes) and waveforms for the cues were calculated with respect to the cue location. For the calculation of the target-related N2pc component we chose the time window of +200 to +300 ms after target onset (+350 to +450 ms after cue onset; see Figure 5). We chose this larger time window due to more variation of the target N2pc compared with the cue N2pc. In this analysis we also included the no-cue condition to control for general effects of the cue display presentation on the target N2pc, which should be apparent in a comparison of the neutral and the no-cue condition.

## Results

### Side Effect Ratings

#### Visual Analog Scale

Results from the side effect ratings are listed in Table 1 and were assessed in a two-way repeated-measures ANOVA with the factors of time (baseline, drug peak, post-testing) and drug (placebo, scopolamine). Analysis of responses from the VAS did not show a significant effect of drug, time or a drug × time interaction for alertness (drug: *F*_(1,26)_ = 1.547, *p* = 0.225; time: *F*_(2,52)_ = 2.384, *p* = 0.102; drug × time: *F*_(2,52)_ = 1.329, *p* = 0.274), contentedness (drug: *F*_(1,26)_ = 3.71, *p* = 0.065; time: *F*_(2,52)_ = 1.195, *p* = 0.311; drug × time: *F*_(2,52)_ = 0.113, *p* = 0.893) or calmness (drug: *F*_(1,26)_ = 3.962, *p* = 0.057; time: *F*_(2,52)_ = 0.392, *p* = 0.678; drug × time: *F*_(2,52)_ = 1.063, *p* = 0.353).

**Table 1:** Side effect ratings from the scopolamine experiment.

	Placebo			Scopolamine		
	Baseline	Peak	Post	Baseline	Peak	Post
**VAS score**
Alertness (%)	42.3 ± 5.9	46 ± 10.8	45.6 ± 9.6	44.5 ± 8.5	45.7 ± 8.8	48.3 ± 12.3
Calmness (%)	31.7 ± 10.1	34.2 ± 11.1	31.8 ± 13.2	36.3 ± 12.2	35.3 ± 12.9	34.8 ± 13.3
Contentedness (%)	38.8 ± 8.9	40.1 ± 9.7	39 ± 11.4	41 ± 11.1	43.4 ± 10.7	41.1 ± 13
**Cardiovascular**
Systolic blood pressure (mmHg)	127 ± 16	124 ± 13	126 ± 12	129 ± 15	122 ± 13	124 ± 11
Diastolic blood pressure (mmHg)	69 ± 9	71 ± 9	73 ± 11	73 ± 9	68 ± 10	70 ± 11
Heart rate (bpm)	74 ± 11	67 ± 8	62 ± 8	76 ± 13	63 ± 15	54 ± 10

*Results for visual analog scale (VAS) and cardiovascular changes over time as a function of drug condition are presented as group mean ± standard deviation. Measurements were taken before drug administration (baseline), at drug peak (peak) and after the contingent capture task (post)*.

#### Cardiovascular Responses

A two-way, repeated-measures ANOVA of systolic blood pressure measures with the factors of time (baseline, drug peak, post-testing) and drug (placebo, scopolamine) showed a significant effect of time (*F*_(2,52)_ = 3.202, *p* = 0.049), but no significant effect of drug (*F*_(1,26)_ = 0.108, *p* = 0.745) or interaction between drug and time (*F*_(2,52)_ = 1.642, *p* = 0.203). The analysis of diastolic blood pressure also showed a significant main effect of time (*F*_(2,52)_ = 4.831, *p* = 0.012), but no significant effect of drug (*F*_(1,26)_ = 0.296, *p* = 0.591). There was however a drug × time interaction (*F*_(2,52)_ = 7.775, *p* = 0.001). *Post hoc* pairwise comparisons with Bonferroni correction revealed that this effect was based on a difference between the placebo (*M* = 69.4 mm Hg, SD = 8.6 mm Hg) and scopolamine (*M* = 72.9 mm Hg, SD = 9.4 mm Hg) condition at baseline (*p* = 0.02), but not at other time points. Heart rate recordings also showed no significant main effect of drug (*F*_(1,26)_ = 2.952, *p* = 0.98), but a significant main effect of time (*F*_(2,52)_ = 65.199, *p* < 0.001). There was a significant time × drug interaction (*F*_(2,52)_ = 10.01, *p* < 0.001). *Post hoc* pairwise comparisons with Bonferroni correction revealed that the interaction was caused by a lower heart rate in the scopolamine condition (*M* = 54.5 bpm, SD = 9.6 bpm) compared with placebo (*M* = 61.59, SD = 8.21 bpm) after testing (*p* < 0.001). At all other time points the measures for the two drugs were not different.

#### Choice Reaction Time Task

Effects of drug treatment on general arousal were tested in a choice reaction time task (see Table 2). Repeated-measures ANOVA with the factors of time (pre-testing, post-testing) and drug (placebo, scopolamine) showed no significant effects on accuracy (drug: *F*_(1,26)_ = 3.12, *p* = 0.089; time: *F*_(1,26)_ = 1.763, *p* = 0.196; drug × time: *F*_(1,26)_ = 0.325, *p* = 0.574) but a significant effect of time on reaction time (*F*_(1,26)_ = 30.043, *p* < 0.001). *Post hoc* pairwise comparisons with Bonferroni correction showed a general slowing of RTs between the first (*M* = 372 ms, SD = 35.1 ms) and second (*M* = 387.7 ms, SD = 32.5 ms) time point. There was, however, no significant effect of drug (*F*_(1,26)_ = 0.309, *p* = 0.583) or a drug × time interaction (*F*_(1,26)_ = 0.191, *p* = 0.665) on RTs.

**Table 2:** Reaction time measure to assess changes in arousal.

	Placebo		Scopolamine	
	Prior drug	Prior testing	Prior drug	Prior testing
Accuracy (%)	97.7 ± 2.1	98.4 ± 2.2	97.3 ± 2.9	97.5 ± 2.2
Reaction time (ms)	371.2 ± 36.3	385.1 ± 29.5	372.9 ± 44.3	390.4 ± 44.4

*Effects of drug treatment on choice reaction times before drug administration (prior drug) and directly before testing (prior testing). Results are displayed as group averages ± standard error*.

### Contingent Capture Performance

The effects of scopolamine, compared with placebo, on performance was assessed by comparing RTs and accuracy for the five different cueing conditions (see Figure 2).

#### Reaction Times

Figure 3A shows RTs as a function of cue and drug conditions. A two-way repeated measures ANOVA on RTs, with the factors of drug (placebo, scopolamine) and cue type (no-cue, neutral, valid, invalid-same-side, invalid-other-side) showed a significant main effect of cue (*F*_(4,104)_ = 95.526, *p* < 0.001), but no effect of drug (*F*_(1,26)_ = 0.173, *p* = 0.681) and no significant cue × drug interaction (*F*_(4,104)_ = 0.743, *p* = 0.565). RTs for valid cues (*M* = 570 ms, SD = 64 ms) were significantly faster than RTs for all other cue conditions (*p* < 0.001, Bonferroni corrected). Neutral cues (*M* = 593 ms, SD = 62 ms) produced significantly longer RTs than valid cues (*p* < 0.001), but RTs were shorter in this condition than in the no-cue condition and the invalid cue condition (*p* < 0.001). There was no difference between the invalid-same-side (*M* = 632 ms, SD = 72 ms) and invalid-other-side condition (*M* = 634 ms, SD = 78 ms). Neither of the invalid cue conditions differed significantly from the no-cue condition (*M* = 629 ms, SD = 62 ms).

#### Accuracy

Figure 3B shows accuracy as a function of cue and drug conditions. A two-way repeated measures ANOVA on the percentage of correct responses with the factors of drug (placebo, scopolamine) and cue type (no-cue, neutral, valid, invalid-same-side, invalid-other-side) showed a significant main effect of cue type (*F*_(4,104)_ = 17.434, *p* < 0.001), as well as an effect of drug (*F*_(1,26)_ = 9.355, *p* = 0.005), but no significant cue × drug interaction (*F*_(4,104)_ = 0.566, *p* = 0.688). *Post hoc* pairwise comparisons with Bonferroni correction showed a general reduction in accuracy for scopolamine trials (*M* = 91.33%, SD = 4.9%) compared with placebo trials (*M* = 93.6%, SD = 4.8%; Cohen’s *d*: 0.42) that was evident in 18 of 27 subjects (67%). Valid cues (*M* = 94.7%, SD = 3.9%) resulted in significantly higher accuracy than both the invalid (*p* < 0.001, Bonferroni corrected) and no-cue conditions (*M* = 93%, SD = 4%; *p* = 0.047), but accuracy was not different from neutral cue trials (*M* = 93.4%, SD = 4.3%). Trials with invalid-same-side cues (*M* = 90.6%, SD = 5.6%) showed significantly decreased accuracy compared with valid, neutral (*p* = 0.002) and no-cue trials (*p* = 0.01). Invalid other side cues (*M* = 90.6%, SD = 5.9%) also showed a decrease in accuracy compared with neutral (*p* = 0.001) and no-cue trials (*p* = 0.01).

### Event Related Potentials

In the analysis of ERPs, we explored the effects of scopolamine on different components related to top-down attention during cue and target processing across the different cue conditions. First we concentrated on the analysis of the P1 and the N1 components, which have been associated with attention-regulated sensory gating (Hopf et al., 2004; Zhang and Luck, 2009). In the next step we analyzed the N2pc component, which is thought to reflect allocation of spatial attention and attention-related enhancement of target information processing (Luck, 2012; Loughnane et al., 2016). In a last step, we analyzed the Pd component, which is indicative of distractor suppression (Sawaki and Luck, 2013).

#### P1 Component—Cue

Figures 4A,B show the topography for target-colored cues compared with neutral cues in the time window +80 to120 ms, which was used in the analysis. We first tested the drug effect on the peak amplitude of the cue-related P1 in the five cue conditions, using a two-way repeated measure ANOVA with the factors of drug (placebo, scopolamine) and cue type (neutral, valid, invalid-same-side, invalid-other-side). In this analysis we found no effect of drug (*F*_(1,26)_ = 0.169, *p* = 0.684) or cue (*F*_(3,78)_ = 0.425, *p* = 0.736) and no interaction between the two factors (*F*_(3,78)_ = 2.65, *p* = 0.055). The same analysis for the peak latency of the cue-related P1 also showed no effect of drug (*F*_(1,26)_ = 0.063, *p* = 0.804), cue (*F*_(3,78)_ = 0.706, *p* = 0.551) or interaction between the two factors (*F*_(3,78)_ = 2.397, *p* = 0.074).

#### P1 Component—Target

To further assess if the target displays showed a different effect on the P1 component than the cue display, we analyzed the peak latency and peak amplitude of the target-display related P1 component in the no-cue condition. In this analysis we used a time window of +80 to 120 ms after target onset. A paired *t*-test of the target P1 under placebo compared with scopolamine was not significant for peak amplitude (*t*_(26)_ = −0.107, *p* = 0.916) or peak latency (*t*_(26)_ = −2.010, *p* = 0.055).

#### N1 Component—Cue

Figures 4C,D show the topography for target-colored cues compared with that for neutral cues in the time window +150 to 200 ms, which was used in this analysis. The grand average waveforms for neutral cues compared with the average of all target-color cues are illustrated in Figure 4E. A two-way repeated measures ANOVA on the peak latency of the cue-related N1 component, with the factors of drug (placebo, scopolamine) and cue (neutral, valid, invalid-same-side, invalid-other-side) showed no significant main effects of drug (*F*_(1,26)_ = 1.909, *p* = 0.179) or cue (*F*_(3,78)_ = 1.482, *p* = 0.226), and no significant interaction between the two factors (*F*_(3,78)_ = 0.244, *p* = 0.865). The same analysis for the peak amplitude of the cue N1 showed a significant main effect of cue (*F*_(3,78)_ = 16.127, *p* < 0.001), but no main effect of drug (*F*_(1,26)_ = 1.034, *p* = 0.319) or drug × cue interaction (*F*_(3,78)_ = 0.506, *p* = 0.679). In *post hoc* pairwise comparisons with Bonferroni correction the peak amplitude of the N1 component for neutral cues (*M* = −6.03 μV, SD = 3.23 μV) was significantly smaller than that for valid (*M* = −6.72 μV, SD = 3.35 μV; *p* < 0.001), invalid-same-side (*M* = −6.58 μV, SD = 3.23 μV; *p* = 0.003) or invalid-other-side (*M* = −6.57 μV, SD = 3.23 μV; *p* < 0.001) cues. None of the amplitudes for the colored-cue conditions were significantly different from each other.

#### N1 Component—Target

We also investigated the latency and peak amplitude of the target N1 in the no-cue condition using a time window of +150 to 200 ms after target onset. A paired *t*-test of the target N1 under placebo compared with scopolamine was not significant for peak amplitude (*t*_(26)_ = −0.37, *p* = 0.715) or peak latency (*t*_(26)_ = 0.428, *p* = 0.672).

#### N2pc Component—Cue

For further assessment of cue-related processing, we investigated the N2pc, time-locked to onset of target-colored cues. Figures 5A,B show the difference waveforms for valid and invalid-same-side cues, and for invalid-other-side cues, respectively. Figures 5D,E show the topographies for left and right target-colored cues. The no-cue condition was excluded from the analysis due to the lack of a cue display and associated responses. Neutral cues were included as a control condition, in which a cue display was presented but no N2pc was expected.

The mean amplitude of the difference waveform in the cue N2pc window for valid, invalid-same-side, invalid-other-side and neutral cues was calculated. A two-way repeated measures ANOVA of the mean amplitude with the factors of cue type (valid, invalid-same-side, invalid-other-side, neutral) and drug (placebo, scopolamine) showed a significant main effect of cue (*F*_(3,78)_ = 22.370, *p* < 0.001), but no main effect of drug (*F*_(1,26)_ = 0.57, *p* = 0.457) or interaction between drug and cue (*F*_(3,78)_ = 1.327, *p* = 0.272). In *post hoc* pairwise comparisons with Bonferroni corrections neutral (*M* = 0.19 μV, SD = 0.56 μV) cues produced significantly smaller amplitudes than valid (*M* = −0.63 μV, SD = 0.93 μV; *p* = 0.001), invalid-same-side (*M* = −0.56 μV, SD = 0.92 μV; *p* < 0.001) or invalid-other-side cues (*M* = −1.18 μV, SD = 1.08 μV; *p* < 0.001). Amplitudes related to invalid-other-side cues were also significantly larger than those for valid (*p* = 0.001) and invalid-same-side cues (*p* < 0.001).

To further investigate potential drug effects on the temporal aspects of the cue N2pc, we analyzed its latency for target-colored cues. A two-way repeated measures ANOVA with the factors drug (placebo, scopolamine) and cue type (valid, invalid-same-side, invalid-other-side) showed no significant effects of drug (*F*_(1,26)_ = 0.032, *p* = 0.859), cue type (*F*_(2,52)_ = 0.938, *p* = 0.389) or interaction between drug and cue type (*F*_(2,52)_ = 0.443, *p* = 0.644) for the peak latency of the cue related N2pc component.

#### N2pc Component—Target

Difference waveforms showing the target N2pc and the associated topography are displayed in Figure 5. A two-way repeated measures ANOVA of target N2pc mean amplitude with the factors drug (placebo, scopolamine) and cue type (valid, invalid-same-side, invalid-other-side, neutral, no-cue) showed a significant main effect of cue (*F*_(4,104)_ = 22.201, *p* < 0.001), but no main effect of drug (*F*_(1,26)_ = 2.968, *p* = 0.097) or drug × cue interaction (*F*_(4,104)_ = 0.589, *p* = 0.671). *Post hoc* pairwise comparisons with Bonferroni corrections showed that invalid-other-side cues (*M* = −1.69 μV, SD = 1.25 μV) produced larger target N2pc amplitudes than the valid (*M* = −0.26 μV, SD = 0.87 μV; *p* < 0.001), invalid-same-side (*M* = −0.56 μV, SD = 0.86 μV; *p* = 0.001), neutral (*M* = −0.61 μV, SD = 0.74 μV; *p* < 0.001) or no-cue (*M* = −0.31 μV, SD = 0.86 μV; *p* < 0.001) conditions. Amplitudes for invalid-same-side cues were only significantly larger than amplitudes for valid cues (*p* = 0.033), but not different from neutral or no-cue conditions. Amplitudes in the neutral cue conditions were also significantly larger than in the no-cue conditions (*p* = 0.05), whereas amplitudes for valid cues were not different from the neutral and no-cue conditions.

A two-way repeated measures ANOVA of target N2pc peak latency with the factors of drug (placebo, scopolamine) and cue type (valid, invalid-same-side, invalid-other-side, neutral, no-cue) showed a significant main effect of cue (*F*_(4,104)_ = 20.432, *p* < 0.001), but no main effect of drug (*F*_(1,26)_ = 0.001, *p* = 0.974) or drug × cue interaction (*F*_(4,104)_ = 0.639, *p* = 0.636). In *post hoc* pairwise comparisons with Bonferroni corrections latencies for invalid-other-side cues (*M* = 388.47 ms, SD = 14.96 ms), neutral cues (*M* = 382.38 ms, SD = 14.81 ms) and no-cue targets (*M* = 386.21 ms, SD = 15.22 ms) were not significantly different from each other. Latencies for valid (*M* = 406.6 ms, SD = 14.39 ms) and invalid-same-side cues (*M* = 404.15 ms, SD = 14.61 ms) were also not significantly different. Latencies for valid cues were significantly longer compared with invalid-other-side (*p* = 0.002), neutral (*p* < 0.001) and no-cues (*p* < 0.001), and the N2pc for invalid-same-side cues was also significantly delayed compared with invalid-other-side (*p* = 0.003), neutral (*p* < 0.001) and no-cues (*p* < 0.001).

#### Cue-Related Pd

The Pd component has been described in relation to attention modulated stimulus processing (Hickey et al., 2009; Sawaki et al., 2012). Since the Pd is thought to reflect visual distractor processing, we were interested in whether the amplitude of the Pd would vary with cue condition within our contingent capture paradigm. Like the N2pc component the Pd is calculated from difference waveforms over posterior lateral electrodes. Inspecting the scalp voltage topography under placebo conditions in the time window of +275 to 325 ms after cue onset (see Figures 5F,G), we used the same electrodes for the calculation of the Pd as for the N2pc. Difference waveforms were calculated by subtraction of ipsilateral from contralateral signals with respect to the target-colored cue location. The neutral cue condition served as a control for effects unrelated to the target-colored cue. Difference waveforms showing the cue Pd are displayed in Figures 5A,B, associated topographies in Figures 5F,G.

A two-way repeated measures ANOVA of the mean Pd amplitude with the factors of drug (placebo, scopolamine) and cue type (valid, invalid-same-side, invalid-other-side, neutral) showed a significant main effect of drug (*F*_(1,26)_ = 5.974, *p* = 0.022; Cohen’s *d* = 0.31) and cue (*F*_(3,78)_ = 27.432, *p* ≤ 0.001), as well as a significant interaction of drug and cue (*F*_(3,78)_ = 5.653, *p* = 0.001). *Post hoc* pairwise comparisons showed a significantly decreased Pd amplitude under scopolamine compared with placebo for valid (*p* = 0.03), invalid-same-side (*p* = 0.037), as well as invalid-other-side cues (*p* = 0.001), but not for neutral cues (*p* = 0.254). Scopolamine-related reductions in the amplitude of the Pd were evident for 19 or 27 (70%) subjects.

Overall, neutral cues (*M* = 0.12 μV, SD = 0.44 μV) produced significantly lower amplitudes than valid (*M* = 1.29 μV, SD = 0.99 μV), invalid-same-side (*M* = 1.33 μV, SD = 0.99 μV) or invalid-other-side (*M* = 0.88 μV, SD = 0.88 μV) cues. Invalid other side cues also produced significantly smaller Pd amplitudes than valid (*p* = 0.03) and invalid-same-side cues (*p* = 0.003), whereas valid and invalid-same-side cue amplitudes were not different from each other. In the invalid-other-side cue condition, no clear Pd peak could be identified as a consequence of a shift of attention from one hemifield to the other and the resulting shift in electrode polarity. Therefore, peak latencies were calculated only for the valid, invalid-same-side and neutral cues.

A two-way repeated measures ANOVA of the Pd peak latency with the factors of drug (placebo, scopolamine) and cue type (valid, invalid-same-side, neutral) showed a significant main effect of cue (*F*_(2,52)_ = 4.345, *p* = 0.018), but no main effect of drug (*F*_(1,26)_ = 0.732, *p* = 0.4) or interaction between drug and cue (*F*_(2,52)_ = 1.556, *p* = 0.221). In *post hoc* pairwise comparisons with Bonferroni correction, the peak latency of the Pd was shorter for neutral (*M* = 300.2 ms, SD = 8.15 ms) compared with invalid-same-side cues (*M* = 304.96 ms, SD = 7.13 ms). Latencies for valid cues (*M* = 304.13 ms, SD = 9.37 ms) were not different from either of the other cue types.

## Discussion

This study investigated the influence of muscarinic-receptor modulation of the top-down control of visual orienting. More specifically, we were interested in the cholinergic modulation of feature-based task set on the processing of task-relevant stimuli as well as distractors. To do so we measured the effect of the muscarinic antagonist *scopolamine* on behavioral and electrophysiological measures of contingent capture (Lien et al., 2008). In the behavioral data, although scopolamine led to an overall decrease in accuracy, there was no indication of a specific cholinergic influence on contingent capture, with participants exhibiting comparable cue validity effects in both conditions. However, the drug did produce a significant attenuation of the distractor-related Pd component compared with placebo.

### Effects of Scopolamine on Performance

Analysis of behavioral data in the placebo condition confirmed that the task did induce a classical contingent capture effect. Specifically, we observed shorter RTs and increased accuracy after presentation of a valid target-colored cue as well as increased RTs and decreased accuracy after presentation of an invalid target-colored cue (Folk et al., 1992; Lien et al., 2008). Despite a non-specific (i.e., cue independent) decrease in accuracy under scopolamine compared with placebo, we did not observe a behavioral modulation of the contingent capture effect by scopolamine. Importantly, scopolamine did not impair performance on the choice reaction time control task, arguing against any simple arousal-based explanation for the overall reduced accuracy under scopolamine. Instead, we suggest that the observed increase in error rate in all conditions of the contingent capture task is related to a disruption of target identification processes. In a previous study in humans using spatial probability to cue the target location, scopolamine decreased the advantage for high probability locations and decreased the disadvantage for low probability locations (Dunne and Hartley, 1986). The latter result was interpreted as an impairment in the ability to allocate attentional capacity to the target location. In another study in monkeys during a simple Posner spatial cueing paradigm, scopolamine specifically decreased the validity effect by increasing the reaction time for valid cues compared with invalid or neutral cues (Davidson et al., 1999). To our knowledge these are the only published studies to examine the impact of systemic administration of scopolamine in combination with a spatial cueing task. Differences in the complexity of cue and target displays makes any direct comparison difficult. Since in the current study we did not find a modulation of the validity effect by scopolamine, we speculate that the overall reduced accuracy we observed after scopolamine administration is the result of an effect of scopolamine on target identification within a complex target display. Thus, scopolamine might decrease the ability to efficiently identify a target amongst distractor stimuli.

### Effects of Scopolamine on Early Visual Components

We further scrutinized electrophysiological responses related to contingent capture to investigate potential effects of scopolamine on top-down attention, which may not have been apparent in the behavioral measures. To investigate the effect of scopolamine during early stimulus processing we measured the P1 and N1 elicited by the cue display. The analysis aimed to identify differences in P1 and N1 amplitudes to target-colored cues compared with neutral cues, and consequently the modulation of these differences by scopolamine. The P1 component was neither modulated by feature-based, top-down attention, nor by scopolamine. The amplitude of the N1, on the other hand, specifically increased in response to presentation of a target-colored cue compared with a neutral cue, as previously shown in a contingent capture paradigm by Arnott et al. (2001). In line with these findings we suggest that the increase in this early visual component reflects a top-down feature-based influence of attention on the processing of the target-colored cue. However, scopolamine did not affect the relative difference in N1 amplitude between target-colored and neutral cue displays. This leads us to the conclusion that scopolamine did not influence top-down influences on early visual components. We suggest that the muscarinic modulation of attention effects in visual cortex, such as those demonstrated in monkey V1, might be restricted to spatial attention (Herrero et al., 2008). However, this suggestion is speculative and requires further experiments in both humans and monkeys to probe the effects of feature-based attention in visual cortex.

### Effects of Scopolamine on N2pc Components

We also measured the N2Pc component as an indicator of contingent capture by stimuli with task relevant features. The N2pc component reflects the allocation of spatial attention toward a selected stimulus. Although the precise function of the N2pc has been a matter of some debate (Eimer, 1996; Eimer and Kiss, 2010; Luck, 2012) it has been consistently linked to the process of target selection. A recent demonstration that the N2pc is observed even in the absence of distractors suggests that it may primarily reflect the enhancement of target features rather than the suppression of distractor information (Mazza et al., 2009; Loughnane et al., 2016).

Under placebo conditions the N2pc following the cue display indicated a classic contingent capture effect/attention shift toward the target-colored cue, as indicated by an increase in amplitude within the N2pc window compared with the neutral condition. We also measured an N2pc in relation to the target display, which represented an attention shift to the target. In line with this notion, the target-N2pc following an invalid-other-side cue displayed the largest amplitude compared with all other cue conditions, indicating that the spatial attention shift between hemifields further enhanced the N2pc.

When we compared the amplitudes and latencies of the cue and target N2pc under placebo with those under scopolamine we found no differences. We conclude that scopolamine did not affect the capturing effect of a target-colored cue, as evident in the cue N2pc, and also did not change the attention shifts towards the target in the target display.

Due to the lack of modulation of the target N2pc, the decrease in accuracy under scopolamine in all cueing conditions cannot be explained by failed or delayed attention shifts toward the target location. Instead, we suggest that scopolamine affected later stages of target processing, occurring after spatial attention is allocated to the target.

### Effects of Scopolamine on the Pd

In the last part of our analysis, we concentrated on a component that followed the cue N2pc, the so-called *posterior positivity* or Pd. This positive deflection in the difference waveform was also observed in the study by Lien et al. (2008), who attributed it to a reversal of the N2pc after the cue display was identified as a distractor (Lien et al., 2008). Although this component was observed in many other previous studies, it was first investigated systematically by Hickey et al. (2009), who named it the posterior positivity, or Pd, and suggested that it reflects the suppression of a distractor stimulus. In their simple discrimination task a Pd was elicited contralateral to an ignored stimulus and was therefore tied to the distractor location. Several subsequent studies confirmed the connection of the Pd with distractor suppression (Sawaki and Luck, 2011, 2013; Hilimire et al., 2012; Kiss et al., 2012; Luck, 2012; Sawaki et al., 2012; McDonald et al., 2013). Hilimire et al. (2012) suggested that the Pd reflects the process of distractor suppression during target disambiguation. Sawaki et al. (2012) further demonstrated that a Pd is elicited during active suppression of attention shifts toward a distractor as well as during reorienting of attention from a target location. Consequently, Sawaki et al. (2012) suggested that the Pd and N2pc might illustrate opposing processes and even share the same neural source in occipital complex and V4. Based on these findings the Pd has been suggested to reflect an active suppression process regulated by top-down attentional processes (Sawaki and Luck, 2013).

In consideration of the close relationship between the N2pc and Pd, as well as the strong dependence of the Pd on top-down control mechanisms, we further investigated the effects of scopolamine on measures of the cue related Pd. We found that scopolamine significantly reduced the Pd amplitude for invalid as well as valid cues. As shown in Figure 5A, in response to valid and invalid-same-side cues the cue N2pc was closely followed by a Pd, which ended with the onset of the target N2pc. The amplitude of this positive deflection was significantly reduced after application of scopolamine compared with placebo. Due to the switch of attention from one hemifield to the other in the invalid-other-side condition, there was a switch in polarity between the cue and the target N2pc in the difference waveforms (see Figure 5B). Therefore, the positive deflection of the cue Pd did not appear as a discrete peak in these waveforms. However, just like in the valid and invalid-same-side condition, the amplitude within the time window of the Pd was significantly reduced under scopolamine.

We suggest that in our study the Pd reflects termination of contingent capture by the target-colored cue after it was identified as a distractor. As described above, this argument is based on the finding that in visual search for a target, which is defined by a conjunction of several features, a distractor with at least one target-defining feature will automatically capture attention, but attention is rapidly withdrawn from the cue (Sawaki et al., 2012; Kiss et al., 2013). This process might be mediated by, or reflected in, the Pd. In line with this argument, the latency of the Pd is thought to depend on the time needed to decide that the item should be suppressed, and is therefore closely related to the complexity of the stimulus as well as the similarity between target and distractor items (Sawaki and Luck, 2011).

Here we have provided evidence that scopolamine significantly reduced the amplitude of the Pd, which suggests reduced suppression of information from the cue display. This effect was consistent across all cueing conditions, but failed to alter the behavioral measures of the effectiveness of the cue display or the different measures of the target N2pc. Reducing the delay period between cue and target, as well as modulating the similarity of cue and targets, might be a good approach to further unmask the effects of scopolamine on distractor processing in future studies.

### Scopolamine and Distractor Suppression

Evidence of a potential mechanism for distractor suppression underlying the Pd was provided by McDonald et al. (2013) who inspected the succession of ERP events related to attentional capture during visual search. They suggested that the sustained posterior contralateral negativity (SPCN), a marker of visual working memory (Jolicœur et al., 2008), was linked to the amplitude of a distractor-related Pd. Distractors that elicited an N2pc and a following Pd did not produce an SPCN. However, there was a trend towards an increase in the SPCN with reduction in the distractor Pd. The authors suggested that the Pd reflects a suppression process, which prevents distractor information from entering working memory. In another study testing the Pd in a delayed match to sample task, distractors presented in the delay period also induced the Pd, if they possessed a target-feature (Sawaki and Luck, 2011). Sawaki and Luck (2011) suggested that a Pd during the distractor display prevented degradation of target-related information in working memory.

Though further research is needed to systematically investigate the causal relationship of Pd and SPCN, a potential association between the Pd component and working memory access is particularly interesting in light of our finding that scopolamine can reduce the Pd for irrelevant information. In an early fMRI study, Furey et al. (2000) demonstrated that administration of the cholinesterase inhibitor physostigmine increased working memory performance during encoding. They concluded that the improvement was based on enhanced perceptual processing selectivity for relevant stimuli in extrastriate visual cortex. Since the increase in ACh levels in response to physostigmine activates nicotinic as well as muscarinic receptors, we cannot conclude that this effect is predominantly mediated by muscarinic receptors. However, more evidence for an involvement of muscarinic modulation in distractor suppression was produced in a study using a flanker paradigm (Thienel et al., 2009). In that study scopolamine increased RTs to incongruent flanker stimuli, which was interpreted as an antagonistic effect on the executive control of attention.

Together, these findings indicate a specific involvement of the cholinergic system in the modulation of selectivity during distractor suppression in late stimulus processing stages. These data are also in line with a dominant view in the literature of an important role for ACh in top-down control, including the filtering of irrelevant information and noise (Sarter et al., 2001, 2005). In consideration of our finding that scopolamine strongly affected a measure of distractor suppression (Pd) but not a measure of attentional capture (N2pc) or top-down stimulus modulation we speculate that previous reports of attentional modulation through the muscarinic pathway might in fact be based on its influence on late stimulus suppression effects. However, further research is needed to specify this interaction. Any such investigations should focus on target-distractor similarities as well as the involvement of working memory. We further anticipate that future research might be able to tie the specific muscarinic effects of top-down control of attention to the more general involvement of the cholinergic system in memory formation (Sarter et al., 2003; Furey, 2011). Such investigations could further include an assessment of muscarinic modulation of intrinsic oscillations. This approach has not been considered in this study but appears promising in light of recent studies investigating the influence of ACh on intrinsic oscillations in relation to memory as well as attention (Bauer et al., 2012; Vakalopoulos, 2014; Eckart et al., 2016).

### Limitations

There are a number of methodological limitations to this study that warrant comment. The first relates to the statistical power of our study. Our sample size of *N* = 30 was adequately powered to observe effects of scopolamine compared with placebo on both behavioral (overall accuracy: Cohen’s *d* = 0.41) and EEG (Pd amplitude: Cohen’s *d* = 0.31) measures. Nevertheless, given that these effect sizes were in the small-medium range, it remains possible that we may have observed drug effects on other variables and contrasts of interest (drug × validity) at higher doses or with larger sample sizes. The second limitation relates to the recruitment of a male-only sample. The decision to exclude female participants was taken to avoid variability in drug pharmacodynamics which may occur across the menstrual cycle. Nevertheless, given known interactions between the cholinergic system and estradiol and progesterone (Gibbs, 2000), this decision should be revisited in future work.

## Summary and Conclusion

Based on previous research which pointed toward involvement of muscarinic receptor activation in the modulation of top-down attention, we investigated the impact of the muscarinic antagonist *scopolamine* on behavioral and electrophysiological markers of contingent capture. Despite strong evidence for top-down modulation of target-features in early stages of processing, we found no evidence for an influence of scopolamine on early visual feature-enhancement or measures of attentional capture by task-relevant features. Instead, we found a consistent reduction in the cue-related Pd component after scopolamine administration. This component has recently been demonstrated to be involved in the suppression of distractor information. We conclude that the previously described influence of ACh on top-down control via muscarinic receptors might be based on a modulation of active suppression of irrelevant distractors. However, the lack of a behavioral correlate of these effects in the current study encourages further research into the involvement of the cholinergic system in distractor processing in humans.

## Author Contributions

IL, NM, AJD, JBM and MAB all contributed to the design of this study. IL, NM and AJD participated in acquisition of data. All authors contributed to the analysis and interpretation of the data. All authors contributed to the preparation of manuscript.

## Conflict of Interest Statement

The authors declare that the research was conducted in the absence of any commercial or financial relationships that could be construed as a potential conflict of interest. The handling editor is currently editing co-organizing a Research Topic with one of the reviewers AD, and confirms the absence of any other collaboration.

## Footnotes

^1^www.neurobs.com
